# The Role of Thoracic Radiation Therapy Dosing in the Treatment of Limited-Stage Small Cell Lung Cancer: A Study Based on the National Cancer Database

**DOI:** 10.1016/j.adro.2022.100907

**Published:** 2022-02-03

**Authors:** Chris Shidal, Evan C. Osmundson, Yong Cui, Hyung-Suk Yoon, Christina E. Bailey, Qiuyin Cai, Xiao-Ou Shu

**Affiliations:** a Division of Epidemiology, Department of Medicine, Vanderbilt Epidemiology Center, Vanderbilt-Ingram Cancer Center, Vanderbilt University School of Medicine, Nashville, Tennessee; b Department of Radiation Oncology, Department of Medicine, Vanderbilt University School of Medicine, Nashville, Tennessee; c Division of Surgical Oncology and Endocrine Surgery, Vanderbilt University Medical Center, Nashville, Tennessee

## Abstract

**Purpose:**

Small cell lung cancer (SCLC) is a highly fatal disease, but its treatment has remained relatively unchanged for decades. Randomized clinical trials evaluating radiation therapy (RT) dosing and fractionation have yielded mixed results on overall survival (OS).

**Methods and Materials:**

We identified 2261 patients with limited-stage (LS) SCLC undergoing definitive RT at 1.5, 1.8, and 2.0 Gy dose per fraction, concurrently with chemotherapy, between 2004 and 2015 within the National Cancer Database. Overall survival (OS) was evaluated using the Kaplan-Meier method, and Cox proportional hazards regression was used to investigate whether there was any survival difference among patients who received hyperfractionated, twice-daily RT at 1.5 Gy per fraction (HF1.5) and once-daily, standard fractionation RT at 1.8 Gy (SF1.8) or 2.0 Gy (SF2.0) per fraction. Subgroup analyses by age, sex, race, time to RT, facility type, and Charlson comorbidity index were also performed.

**Results:**

All stage median OS rates for HF1.5, SF1.8, and SF2.0 Gy groups were 21.6, 18.9, and 19.4 months, respectively (log-rank *P* = .0079). Multivariate analyses adjusting for demographic factors, socioeconomic status, tumor characteristics, and year of diagnosis showed SF1.8 (hazard ratio [HR] = 1.30, 1.03-1.63) and SF2.0 (HR = 1.20, 1.00-1.45) was associated with worse 1-year survival compared with HF1.5. This association was more evident in stage IIb-stage III than stage I to stage IIa patients. Propensity score–weighted analysis showed similar results. Stratified analyses showed the significant associations were confined to male or black patients, those aged >65 years, with 1 comorbidity, who had waited >60 days to start RT or were treated at an academic medical center.

**Conclusions:**

Analyses of real-world treatment outcome data showed that receiving hyperfractionated, twice-daily RT was associated with improved survival among patients with LS-SCLC compared with standard, once-daily fractionation regimens at 1 year after diagnosis, particularly for subsets of patients. Some associations retained statistical significance 3 years postdiagnosis.

## Introduction

Although accounting for only 15% of all lung cancer cases, small cell lung cancer (SCLC) is among the most aggressive malignancies, with 5-year survival rates of 27%, 16%, and 3% for localized, regional, and distant stages, respectively.^1^ Therapeutic options to treat both limited-stage (LS) and extensive-stage (ES) SCLC have remained relatively unchanged since the 1970s.^2^ The current National Comprehensive Cancer Network (NCCN) recommendation for the treatment of SCLC is thoracic radiation therapy (RT) in combination with concurrent chemotherapy.^3^ The addition of RT to chemotherapy was shown to moderately improve LS-SCLC survival in a comprehensive meta-analysis.^4^ Additionally, surgical resection followed by adjuvant chemotherapy has been reported to improve survival in patients with LS-SCLC without nodal involvement and may present a superior treatment strategy compared with concurrent chemoradiotherapy (CCRT) in these highly selected cases.^5^, ^6^, ^7^, ^8^, ^9^ Per NCCN guidelines, CCRT is preferred when surgery is not indicated.^3^

A focus for recent investigations is whether RT dosing and fractionation influence SCLC survival outcomes.^10^, ^11^, ^12^ The Concurrent ONce-daily VErsus twice-daily RadioTherapy (CONVERT) trial investigated whether a twice-daily (bid) schedule (45 Gy/30 fractions) provided any benefit to patients with LS-SCLC compared with a once-daily (QD) schedule (66 Gy/33 fractions). This phase III randomized trial showed that patients receiving daily RT or hyperfractionated RT had similar survival outcomes.^13^ The ongoing CALGB30610 trial compares LS-SCLC survivorship among 3 treatment arms consisting of hyperfractionated (45 Gy/30 fractions), daily (70 Gy/35 fractions), and accelerated (61.2 Gy/34 fractions) fractionation regimens. The accelerated fractionation arm was discontinued in 2012 for logistical reasons, and mature results are not expected until 2023.^14^ Interim CALGB30610 data showed no significant differences between once-daily and hyperfractionated treatment arms.^15^

In the current study, we analyzed real-world treatment outcome data from the National Cancer Database (NCDB) to evaluate whether different RT doses, that is, twice-daily RT at 1.5 Gy per fraction (HF1.5) and standard fractionated, once-daily courses using 1.8 Gy (SF1.8) and 2.0 Gy (SF2.0) doses per fraction, were associated with OS in patients with LS-SCLC undergoing CCRT. We further performed stratified analyses to determine whether any of the RT regimens were associated with a survival benefit within subgroups of patients.

## Materials and Methods

### Study design and variable construction

The NCDB is a clinical oncology database that represents >70% of all newly diagnosed cancer cases in the United States. Variables collected include demographics information, tumor characteristics, and treatment data, among several others. A full description of the NCDB study design and data collection has been described elsewhere.^16^^,^^17^ In our study, a total of 238,691 individuals diagnosed with SCLC between 2004 and 2015 were identified. We excluded individuals with missing cancer staging data (n = 19,856), who received treatment other than CCRT (n = 162,952), and who received RT at a site other than the lungs/chest (n = 20,437). We additionally excluded patients who received palliative RT (n = 14,439) or had missing and other volume/dosing information (n = 7606). Furthermore, we focused only on participants receiving between 30 and 35 total doses of definitive RT at dose-per-fraction values of 1.5 Gy (n = 876), 1.8 Gy (n = 393), and 2.0 Gy (n = 992). Standard fractionation (SF1.8 and SF2.0) doses were limited to a total dose of 60 to 70 Gy. To eliminate the accelerated dosing regimen (ie, concomitant boost), the HF1.5 group was restricted to patients completing RT within 19 to 23 days after RT initiation; once-daily SF1.8 and SF2.0 groups were restricted to completing RT within 44 to 54 days. This range of values represented a 10% deviation from the exact number of days in the treatment plan (45 Gy in 30 fractions for 21 days or 60-70 Gy in 30-35 fractions for 49 days to accommodate missed doses, patients completing >90% of RT, and to exclude patients with prolonged or discontinued RT). The NCDB does not contain information on RT fractionation; thus, patients in the 1.5 Gy group were assumed to be on a hyperfractionated schedule.

### Statistical analysis

Descriptive variables were compared among treatment regimens, and *P* values were derived using the Student *t* test for continuous variables or χ^2^ test for categorical variables. Kaplan-Meier univariate analysis was performed to estimate overall survival (OS) among treatment groups, and a log-rank test was performed to determine statistical significance among groups. Multivariate analysis using a Cox proportional hazards model was conducted to estimate hazard ratios (HR) and 95% confidence intervals (CI) among groups. The following variables were adjusted for: age (continuous), sex (male, female), race (white, black, other), neighborhood education (percent with no high school diploma; ≥29, 20-28.9, 14-19.9, <14) and income quartile (1, 2, 3, 4), insurance (uninsured, private, Medicare, Medicaid, other government, unknown), facility type (academic, community, comprehensive, integrated, other), facility location (rural, urban, metro), distance to facility (miles; ≤50, 50-100, >100), Charlson comorbidity index (0, 1, ≥2), TNM stage (T0-4, N0-3), year of diagnosis (continuous), and time to RT from diagnosis (≤30 days, 30-60 days, ≥60 days). Survival estimates for 1- and 3-year intervals were estimated from univariate and multivariate analyses. We derived propensity scores (PS) to estimate the probability of receiving different RT doses/fractionations, conditioned on all possible confounders. We then performed PS-weighted analyses using the inverse PS method, adjusting for the sample size proportion of each treatment group in the Cox regression, accordingly.

Stratified analyses were conducted to examine whether the RT dose and mortality association was modified by patients’ disease, treatment, and demographic characteristics. Stratification by tumor stage was performed by stage I-stage IIa and stage IIb-stage III. The Veterans Administration Lung Study Group (VALG) criteria, which remain controversial, is traditionally used to classify SCLC into LS or ES.^18^ Our categorization by TNM criteria was performed to allow additional interpretations, based on tumor characteristics that were consolidated using the VALG definitions, and reflects stage-specific management algorithms used in clinical practice and recommended NCCN guidelines.^3^ Statistical significance was set at *P* < .05. All statistical analyses were performed using SAS Enterprise Guide 7.1 (Cary, NC).

## Results

A total of 2261 LS-SCLC cases were included in the study. Comparisons of demographic variables among treatment groups are presented in Table 1. Patients in the HF1.5 group were younger than the SF1.8 and SF2.0 groups (*P* < .0001). Significant differences were observed for insurance status (*P* < .0001), but not income (*P* = .25) and education (*P* = .14). No differences were observed in race (*P* = .38) or sex (*P* = .48). Patients receiving SF1.8 or SF 2.0 tended to be covered under Medicare or Medicaid at higher rates than those receiving hyperfractionated RT; individuals receiving HF1.5 were more likely to be covered under private insurance. The HF1.5 group was more likely to receive treatment at an academic facility and travel farther for treatment compared with SF1.8 and SF2.0 groups, and the latter were more likely to receive treatment in community or comprehensive cancer centers. No difference was observed for facility location (*P* = .20). Tumor characteristics, including tumor size, node positivity and stage, were similar among RT groups. The HF1.5 group had a lower comorbidity score (*P* < .0001) and received RT more quickly (≤60 days of diagnosis; *P* < .0001) compared with the SF1.8 or SF2.0 groups.

**Table 1:** Demographics of the study participants

	1.5HF	1.8SF	2.0SF	*P* value*
	n = 876	n = 393	n = 992	
**Age**	62.2 ± 9.3	64.0 ± 9.6	64.3 ± 9.9	**<.01**
**Sex**				
Male	418 (47.7)	184 (46.8)	446 (45.0)	
Female	458 (52.3)	209 (53.2)	546 (55.0)	.48
**Race**				
Black	67 (7.7)	38 (9.7)	92 (9.3)	
White	781 (89.2)	344 (87.5)	879 (88.6)	
Other/unknown	28 (3.1)	11 (2.8)	21 (2.1)	.38
**Insurance status**				
Uninsured	43 (4.9)	13 (3.3)	36 (3.6)	
Private	348 (39.7)	135 (34.4)	302 (30.4)	
Medicare	386 (44.1)	209 (53.2)	525 (52.9)	
Medicaid	66 (7.5)	32 (8.1)	107 (10.8)	
Government (other)	22 (2.5)	7 (0.6)	12 (1.2)	
Unknown	11 (1.3)	1 (0.3)	10 (1.0)	**<.01**
**Facility type**				
Academic	433 (49.4)	97 (24.7)	356 (35.9)	
Community	64 (7.3)	56 (14.3)	127 (12.8)	
Comprehensive	312 (35.6)	197 (50.1)	421 (42.4)	
Integrated	59 (6.7)	41 (10.4)	79 (8.0)	
Missing	8 (0.9)	2 (0.5)	9 (0.9)	**<.01**
**Income (quartile)**				
1	189 (21.6)	70 (17.8)	207 (20.9)	
2	225 (25.7)	111 (28.2)	274 (27.6)	
3	229 (26.1)	97 (24.7)	279 (28.1)	
4	221 (25.2)	106 (27.0)	221 (22.3)	
Missing	12 (1.4)	9 (2.3)	11 (1.1)	.25
**Education**				
**(% no HSD)**				
≥29	172 (19.6)	60 (15.3)	218 (22.0)	
20-28.9	230 (26.3)	118 (30.0)	270 (27.2)	
14-19.9	287 (32.8)	134 (34.1)	306 (30.9)	
<14	175 (20.0)	72 (18.3)	187 (18.9)	
Missing	12 (1.4)	9 (2.3)	11 (1.1)	.14
**Facility location**				
Metro	677 (77.3)	305 (77.6)	755 (76.1)	
Urban	173 (19.8)	84 (21.4)	204 (20.6)	
Rural	26 (3.0)	4 (1.0)	33 (3.3)	.2
**Distance to facility, miles**				
≤50	743 (84.8)	360 (91.6)	929 (93.7)	
50-100	78 (8.9)	28 (7.1)	51 (5.1)	
>100	55 (6.3)	5 (1.3)	12 (1.2)	**<.01**
**Charlson-Deyo Comorbidity Index**				
0	572 (65.3)	229 (58.3)	566 (57.1)	
1	236 (26.9)	106 (27.0)	284 (28.6)	
≥2	68 (7.8)	58 (14.8)	142 (14.3)	**<.01**
**TNM (T)**				
0	9 (1.0)	2 (0.5)	7 (0.7)	
1	211 (24.1)	84 (21.4)	241 (24.3)	
2	268 (30.6)	120 (30.5)	325 (32.8)	
3	125 (14.3)	57 (14.5)	152 (15.3)	
4	209 (23.9)	101 (25.7)	227 (22.9)	
Unknown	54 (6.2)	29 (7.4)	40 (4.0)	.32
**TNM (N)**				
0	140 (16.0)	65 (16.5)	178 (17.9)	
1	104 (11.9)	41 (10.4)	122 (12.3)	
2	497 (56.7)	210 (53.4)	531 (53.5)	
3	115 (13.1)	64 (16.3)	139 (14.0)	
Unknown	20 (2.3)	13 (3.3)	22 (2.2)	.6
**AJCC stage**				
I	82 (9.4)	37 (9.4)	101 (10.2)	
II	100 (11.4)	38 (9.7)	134 (13.5)	
III	694 (79.2)	318 (80.9)	757 (76.3)	.27
**Laterality**				
Midline, unpaired, or unknown	88 (10.1)	51 (13.0)	104 (10.5)	
Right	459 (52.4)	215 (54.7)	530 (53.4)	
Left	329 (37.6)	127 (32.3)	358 (36.1)	.33
**Days to RT from Dx**				
≤30	290 (33.1)	126 (32.1)	268 (27.0)	
30-60	402 (45.9)	147 (37.4)	408 (41.1)	
>60	184 (21.0)	119 (30.3)	313 (31.6)	
Missing	0 (0.0)	1 (0.3)	3 (0.3)	**<.01**

*Abbreviations:* AJCC = American Joint Committee on Cancer; Dx = diagnosis; HSD = high school diploma; RT = radiation therapy; SF = standard fractionation.

⁎ An analysis of covariance was used to investigate differences among RT groups by the *t* test procedure for continuous variables and χ^2^ test for categorical variables.

**Fig. 1:**
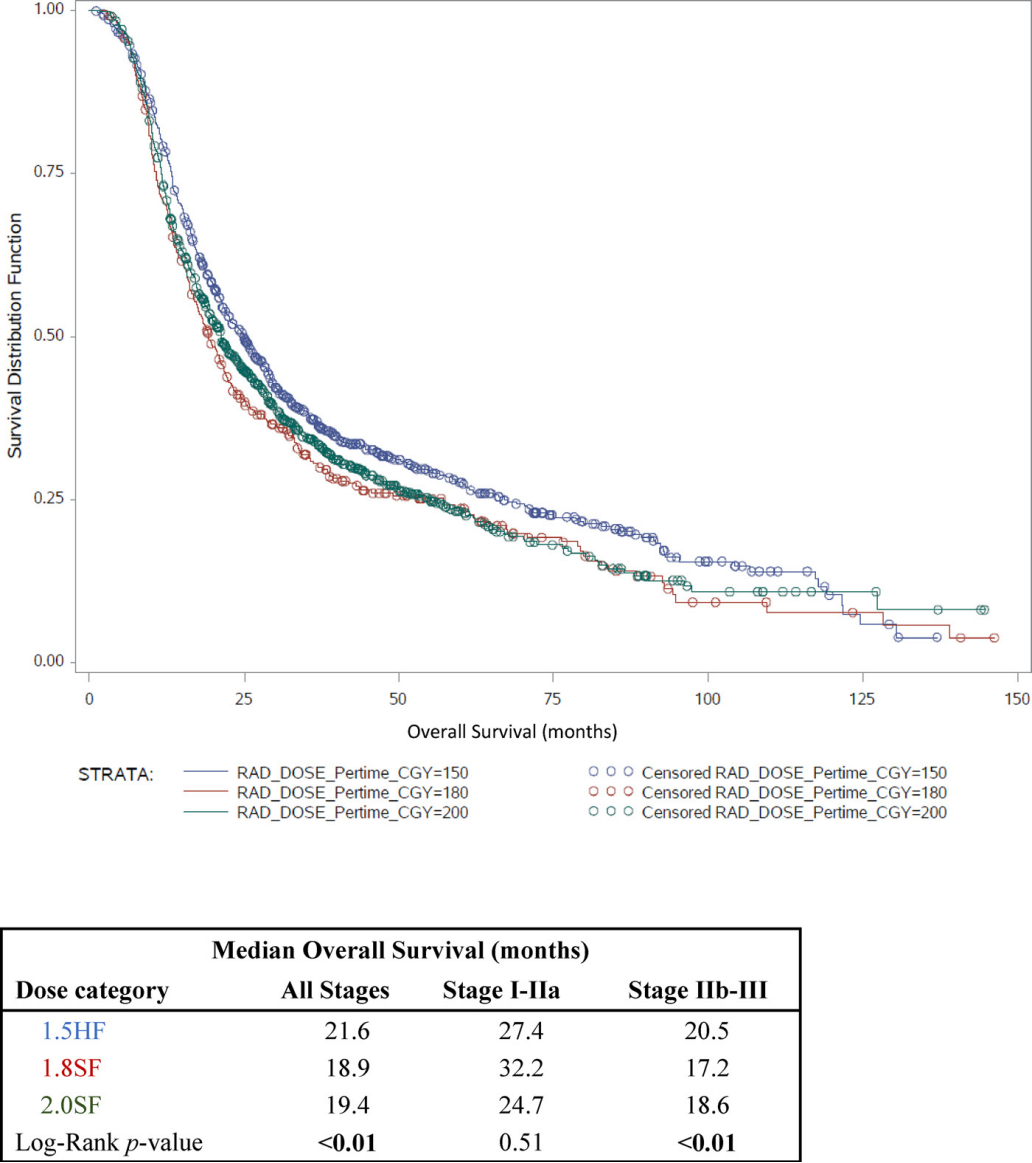
Kaplan-Meier estimates for overall survival (OS) by radiation therapy (RT) dose. Kaplan-Meier survival curves are shown for HF1.5 (blue), SF1.8 (red), and SF2.0 (green) RT treatment groups. The surviving fraction (y-axis) is represented as a function of time (x-axis, in months). Censored events are represented by the open circles Table 1. shows median OS (in months) for combined stage, stage I-IIa, and stage IIb-III limited stage small cell lung cancer patients. A log-rank test was performed to compare median survival among RT groups, and the resulting *P* values are shown. *Abbreviation:* RAD = radiation.

Univariate analysis showed significant differences in median survival times among RT groups: 21.6, 18.9, and 19.4 months for HF1.5, SF1.8, and SF2.0 groups, respectively (Fig. 1). Significant differences in median survival times among RT groups were observed in all stages combined (*P* = .0079) and stage IIb-III (*P* = .0094), but not in stage I-IIa (*P* = .5123). Results of univariate analysis, comparing 1- and 3-year survival rates among RT groups, are shown in Table 2. Among all cases, 1-year survival rates were 76.3%, 66.9%, and 69.3% for HF1.5, SF1.8, and SF2.0 groups, respectively (*P* = .0007). A significant difference in 1-year survival was observed in stage IIb-III (*P* = .0009), but not in stage I-IIa (*P* = .5966) patients. In all cases, 3-year survival rates for HF1.5, SF1.8, and SF2.0 groups were 40.4%, 32.6%, 38.4%, respectively (*P* = .0116). A significant difference in 3-year survival among RT groups was observed in stage IIb-III (*P* = .0041), but not in stage I-IIa (*P* = .505) SCLC.

**Table 2:** 1- and 3-year survival rates by radiation therapy dose

Survival rates (%)		
	1-Year	3-Year
**Stage I-IIa**		
1.5HF	83.1	51.4
1.8SF	80	54.6
2.0SF	78.5	48.7
Log-rank *P*	.6	.51
**Stage IIb-III**		
1.5HF	74.9	38.2
1.8SF	64.8	28.9
2.0SF	67.1	35.9
Log-rank *P*	**<.01**	**<.01**
**All cases**		
1.5HF	76.3	40.4
1.8SF	66.9	32.6
2.0SF	69.3	38.4
Log-rank *P*	**<.01**	**.01**

*Abbreviations:* SF = standard fractionation; HF = hyperfractionated

Hazard ratios (HRs) for overall, 1-, and 3-year mortality adjusting for age, race, sex, education, income, insurance, facility type, facility location, distance to facility, year of diagnosis, primary tumor size, spread to lymph nodes, comorbidity index, and days to radiation from diagnosis are shown in Table 3. Overall, we observed HRs of 1.12 (0.97-1.30) and 1.04 (0.93-1.17) for SF1.8 and SF2.0 groups, respectively, compared with the HF1.5 group. HRs for SF1.8 (HR = 1.16, 0.99-1.36) and SF2.0 (HR = 1.06, 0.93-1.20) compared with HF1.5 in stage IIb-III patients did not reach statistical significance. Similarly, no significant associations between RT group and overall mortality were observed for stage I-IIa SCLC. However, analysis of 1-year survival showed that HRs for SF1.8 and SF2.0 RT groups were significantly higher compared with the HF1.5 group (SF1.8 HR = 1.30, 1.03-1.63; SF2.0 HR = 1.20, 1.00-1.45). This association was observed in stage IIb-stage III SCLC, but not in stage I-IIa SCLC. Compared with 1.5HF, 1.8SF was associated with worse 3-year survival in patients with stage IIb-III SCLC (HR = 1.21, 1.03-1.43), but not in all (HR = 1.14, 0.98-1.33) or stage I-IIa (HR = 0.76, 0.47-1.25) SCLC cases. No significant association was observed in 3-year survival comparing SF2.0 with the hyperfractionated RT group. PS-weighted analyses provided results that mirrored multivariate analyses (Table 3).

**Table 3:** Hazard ratios for 1-year, 3-year, and overall survival by radiation therapy dose

	Overall		1-Year		3-Year	
	Death/total	HR (95% CI)	Death/total	HR (95% CI)	Death/total	HR (95% CI)
Multivariate*						
**All cases**						
1.5 Gy	601/866	1.00 (ref)	205/866	1.00 (ref)	516/866	1.00 (ref)
1.8 Gy	299/387	1.12 (0.97-1.30)	128/387	**1.30 (1.03-1.63)**	261/387	1.14 (0.98-1.33)
2.0 Gy	682/983	1.04 (0.93-1.17)	302/983	**1.20 (1.00-1.45)**	606/983	1.04 (0.92-1.18)
**Stage I-IIa**						
1.5 Gy	94/148	1.00 (ref)	25/148	1.00 (ref)	72/148	1.00 (ref)
1.8 Gy	40/55	0.98 (0.64-1.48)	11/55	0.99 (0.46-2.14)	25/55	0.76 (0.47-1.25)
2.0 Gy	124/191	1.02 (0.76-1.37)	41/191	1.04 (0.61-1.80)	98/191	0.99 (0.71-1.38)
**Stage IIb-III**						
1.5 Gy	507/718	1.00 (ref)	180/718	1.00 (ref)	444/718	1.00 (ref)
1.8 Gy	259/332	1.16 (0.99-1.36)	117/332	**1.31 (1.03-1.67)**	236/332	**1.21 (1.03-1.43)**
2.0 Gy	558/792	1.06 (0.93-1.20)	261/792	**1.22 (1.00-1.49)**	508/792	1.06 (0.93-1.22)
*P-*interaction		0.67		0.86		0.17
**Propensity score–weighted**†						
**All cases**						
1.5 Gy (n = 876)	601/866	1.00 (ref)	205/866	1.00 (ref)	516/866	1.00 (ref)
1.8 Gy (n = 393)	299/387	1.14 (0.99-1.31)	128/387	**1.35 (1.08-1.69)**	261/387	1.13 (0.97-1.31)
2.0 Gy (n = 992)	682/983	1.05 (0.94-1.17)	302/983	**1.21 (1.01-1.44)**	606/983	1.05 (0.93-1.18)
**Stage I-IIa**						
1.5 Gy (n = 148)	94/148	1.00 (ref)	25/148	1.00 (ref)	72/148	1.00 (ref)
1.8 Gy (n = 55)	40/55	0.91 (0.63-1.32)	11/55	1.02 (0.50-2.07)	25/55	0.73 (0.46-1.15)
2.0 Gy (n = 191)	124/191	1.02 (0.78-1.33)	41/191	1.19 (0.73-1.93)	98/191	0.99 (0.74-1.34)
**Stage IIb-III**						
1.5 Gy (n = 718)	507/718	1.00 (ref)	180/718	1.00 (ref)	444/718	1.00 (ref)
1.8 Gy (n = 332)	259/332	**1.17 (1.01-1.36)**	117/332	**1.37 (1.08-1.73)**	236/332	**1.19 (1.01-1.39)**
2.0 Gy (n = 792)	558/792	1.05 (0.93-1.18)	261/792	**1.20 (1.00-1.46)**	508/792	1.05 (0.93-1.20)

*Abbreviations:* CI = confidence interval; HR = hazard ratio.*Note:* Figures in bold represent statistically significant Hazard Ratios

⁎ Adjusted for age, sex, race, education, income, insurance, facility type, facility location, distance to facility, comorbidity, year of diagnosis, time to radiation therapy, and TNM stage.

† Using a propensity score–weighted Cox regression model.

No significant associations between RT group and OS were observed when stratified by sex, race, or comorbidity status in overall and 3-year mortality (Table 4). In overall analyses, SF1.8 was associated with worse OS compared with HF1.5 in patients >65 years (HR = 1.39, 1.12-1.72). When RT was administered >60 days following diagnosis, OS was significantly worse in SF groups, that is, 1.8 Gy (HR = 1.81, 1.37-2.39) and 2.0 Gy (HR = 1.29, 1.02-1.63) compared with the HF1.5 group. SF1.8 was associated with worse survival when treatment was initiated at an academic treatment facility (HR = 1.47, 1.13-1.92). Additional analyses of 1-year mortality stratified by these covariates showed that SF1.8 was associated with significantly increased 1-year mortality among patients >65 years (HR = 1.60, 1.16-2.19), male patients (HR = 1.44, 1.05-1.99), African Americans (HR = 3.07, 1.07-8.80), patients with a comorbidity (comorbidity score = 1, HR = 1.67, 1.09-2.57), those with days to RT >60 days (HR = 2.56, 1.57-4.17), or those treated in an academic setting (HR = 2.21, 1.46-3.35). Similar association patterns were observed for the 2.0SF RT group. Analysis for 3-year mortality showed similar trends, although the point estimates were attenuated among male patients, African Americans, and cases with a comorbidity. The significant associations between 3-year mortality and RT group remained for individuals >65 years of age, those who waited >60 days from diagnosis to RT, and those treated in an academic treatment facility. Notably, we observed significant interactions between RT group and treatment facility type and between RT group and days to RT from diagnosis.

**Table 4:** Risk of death at 1-year, 3-year, and overall stratified by covariates*

	HR (95% CI) overall survival			HR (95% CI) 1-year survival			HR (95% CI) 3-year survival		
Age	1.5HF	1.8SF	2.0SF	1.5HF	1.8SF	2.0SF	1.5HF	1.8SF	2.0SF
≤65	1.00	1.00 (0.82-1.21)	1.07 (0.92-1.25)	1.00	1.10 (0.78-1.54)	1.26 (0.97-1.64)	1.00	0.98 (0.79-1.21)	1.08 (0.91-1.27)
>65	1.00	**1.39 (1.12-1.72)**	1.14 (0.95-1.35)	1.00	**1.60 (1.16-2.19)**	1.28 (0.98-1.67)	1.00	**1.42 (1.13-1.78)**	1.10 (0.91-1.32)
*P-*interaction	0.12			0.25			0.09		
**Sex**									
Male	1.00	1.14 (0.93-1.41)	1.05 (0.89-1.24)	1.00	**1.44 (1.05-1.99)**	**1.29 (1.00-1.68)**	1.00	1.17 (0.94-1.46)	1.04 (0.87-1.24)
Female	1.00	1.14 (0.94-1.40)	1.05 (0.89-1.24)	1.00	1.14 (0.94-1.40)	1.05 (0.89-1.24)	1.00	1.17 (0.94-1.45)	1.05 (0.89-1.25)
*P-*interaction	0.92			0.99			0.93		
**Race**									
White	1.00	1.09 (0.94-1.27)	1.04 (0.92-1.17)	1.00	1.26 (0.99-1.61)	1.20 (0.99-1.46)	1.00	1.13 (0.96-1.33)	1.04 (0.92-1.19)
Black	1.00	1.73 (0.99-3.04)	1.51 (0.94-2.43)	1.00	**3.07 (1.07-8.80)**	**2.45 (1.00-5.97)**	1.00	1.62 (0.89-2.94)	1.45 (0.89-2.37)
*P-*interaction	0.81			0.46			0.91		
**Charlson-Deyo Comorbidity Index**									
0	1.00	1.08 (0.90-1.30)	1.08 (0.93-1.25)	1.00	1.16 (0.85-1.59)	1.16 (0.91-1.48)	1.00	1.10 (0.90-1.35)	1.11 (0.94-1.30)
1	1.00	1.20 (0.90-1.59)	1.11 (0.89-1.38)	1.00	**1.67 (1.09-2.57)**	**1.51 (1.06-2.13)**	1.00	1.24 (0.93-1.67)	1.10 (0.88-1.39)
≥2	1.00	1.07 (0.69-1.68)	0.96 (0.65-1.43)	1.00	1.29 (0.68-2.46)	1.12 (0.63-1.97)	1.00	1.07 (0.68-1.70)	0.85 (0.56-1.28)
*P-*interaction	0.42			0.75			0.23		
**Time to RT from diagnosis**									
<30 days	1.00	1.10 (0.85-1.43)	0.88 (0.71-1.09)	1.00	1.18 (0.79-1.76)	1.03 (0.74-1.42)	1.00	1.14 (0.86-1.50)	0.83 (0.66-1.05)
30-60 days	1.00	0.82 (0.65-1.05)	1.06 (0.89-1.27)	1.00	1.00 (0.68-1.45)	1.12 (0.84-1.49)	1.00	0.85 (0.66-1.10)	1.09 (0.90-1.31)
>60 days	1.00	**1.81 (1.37-2.39)**	**1.29 (1.02-1.63)**	1.00	**2.56 (1.57-4.17)**	**2.07 (1.34-3.21)**	1.00	**1.83 (1.36-2.45)**	**1.33 (1.03-1.71)**
*P-*interaction	0.05			0.07			**0.03**		
**Facility type**									
Academic	1.00	**1.47 (1.13-1.92)**	1.18 (0.98-1.41)	1.00	**2.21 (1.46-3.35)**	**1.47 (1.07-2.00)**	1.00	**1.47 (1.10-1.96)**	1.21 (0.99-1.47)
Community	1.00	1.13 (0.71-1.80)	1.17 (0.79-1.75)	1.00	1.12 (0.54-2.30)	1.10 (0.60-2.03)	1.00	1.00 (0.62-1.62)	1.08 (0.72-1.62)
Comprehensive	1.00	0.89 (0.72-1.09)	0.86 (0.72-1.03)	1.00	0.96 (0.69-1.32)	0.97 (0.74-1.28)	1.00	0.92 (0.74-1.15)	0.85 (0.70-1.03)
Integrated	1.00	1.02 (0.55-1.89)	1.31 (0.78-2.22)	1.00	0.74 (0.28-1.97)	0.95 (0.42-2.17)	1.00	1.07 (0.55-2.08)	1.23 (0.69-2.20)
*P-*interaction	**0.04**			**0.03**			0.14		

*Abbreviation:* CI = confidence interval; HR = hazard ratio; RT = radiation therapy; SF = standard fractionation.

⁎ Adjusted for age, sex, race, education, income, insurance, facility type, facility location, distance to facility, comorbidity, year of diagnosis, time to radiation therapy, and TNM stage.

## Discussion

In our retrospective study analyzing real-world treatment and outcome data from the NCDB, we found that patients with LS-SCLC who received daily, standard fractionations of 1.8 Gy and 2.0 Gy per fraction had worse survival compared with those who received hyperfractionated RT at 1.5 Gy. After adjustment for patient demographics, socioeconomic status, treatment-related variables, and tumor characteristics, treatments with SF1.8 and SF 2.0 were associated with significantly worse 1-year survival among all cases and patients with stage IIb-III SCLC compared with those treated with HF1.5. The 3-year survival was significantly worse in stage IIb-III SCLC patients treated with the SF 1.8 regimen compared with the HF1.5 regimen. PS-weighted regression showed similar results to those from multivariate analyses. Taken together, these results suggest that more patients with locally advanced, LS-SCLC, treated with bid fractionation, survive to 1- and 3-year intervals, even though they may ultimately succumb to their disease, at rates comparable to their counterparts treated on daily fractionation regimens. However, in a disease with as poor of a prognosis as SCLC, these improvements in short-term survival are meaningful for patients and their families. Stratified analyses indicate that the associations between OS and RT treatments were modified by patient characteristics such as age, sex, race, and comorbidity status, with significant associations primarily seen among older (>65 years), black, or male individuals. These significant associations were primarily seen in SCLC patients waiting longer (>60 days) to initiate treatment and in patients treated at academic treatment facilities.

In 1999, Turrisi et al reported that hyperfractionated thoracic RT considerably improved survival in a trial involving more than 400 SCLC patients compared with the once-daily RT regimen.^12^ However, this study has been criticized for using the same total dose (45 Gy) for each treatment schema, which reduced the biologically effective dose (BED) in the daily treatment compared with the bid treatment. A pooled analysis of CALGB trials using escalated daily RT dosing to a more comparable BED (70 Gy over 35 fractions) demonstrated that this dose was well tolerated, and OS was comparable to hyperfractionated RT (total dose of 45 Gy) in patients with LS-SCLC.^19^ Interestingly, a study by Rutter et al reported that doses >70 Gy did not provide additional improvement in LS-SCLC survival for total doses of 45 to 70 Gy.^20^ Similarly, high-dose RT (74 Gy) did not improve OS compared with standard dose RT (60 Gy) in a randomized, phase III trial of stage III non-SCLC patients undergoing CCRT and was suspected to result in increased treatment-related deaths from toxicity.^21^ More recently, a randomized phase II trial of patients with LS-SCLC demonstrated that higher total doses of hyperfractionated RT (60 Gy in 40 bid fractions) yielded significantly improved survival compared with 45 Gy for 30 fractions, without a significant increase in dose-related toxicity.^22^ These data, taken together, illustrate the progression of studies aiming to optimize RT timing and dosing in LS-SCLC.

Evidence indicates timing of RT initiation may be of substantial importance. A study by Wong et al indicated that RT fractionation and initiation of RT relative to the start of chemotherapy may affect survival.^23^ The results of the current study are in agreement with the findings from Wong and colleagues that showed a significant association between improved survival and twice-daily RT at a total dose of 45 Gy when compared with once-daily fractionation. Wong et. al. also showed that earlier initiation of the hyperfractionated RT regimen was associated with improved survival; however, our study did not assess the timing component with respect to chemotherapy initiation.

Significant associations between RT dosing and survival were limited to stage IIb-III SCLC in our population; no statistically significant associations were observed in analyses of stage I-IIa SCLC patients. Although chemoradiation is commonly used for LS-SCLC treatment, NCCN guidelines recommend surgical resection of T1-2N0M0 SCLC in patients who can tolerate it. More recent publications suggest that the use of stereotactic ablative radiation therapy (SABR), followed by consolidative doublet chemotherapy, may provide additional benefit compared with CCRT in unresectable, node-negative stage I-IIa SCLC. Consistent with NCCN recommended management paradigms, we, therefore, categorized patients with LS-SCLC into 2 groups: stage I-IIa and stage IIb-III. Additionally, stage IIb encompasses a relatively heterogeneous population (eg, T4N0M0 vs T1N1M0), which could potentially diminish our ability to detect true associations in this subset of patients. Conversely, our sample size, which was limited by stringent inclusion criteria and high response rates of early-stage SCLC to initial CCRT, may explain the absence of a significant association when restricting analyses to stage I-IIa patients in our study.

Stratified analyses identified significant disparities in survival among subsets of patients receiving different RT regimens. Although the technology for advanced RT (ie, intensity-modulated RT) has existed for nearly 2 decades, not all facilities have these capabilities, which may explain the differences observed comparing RT groups; hyperfractionated RT was also more likely to be administered at academic centers, which may have improved logistical capacity for bid treatment and provide the ancillary support for patients undergoing this more intensive treatment. Although our subcohort had relatively low comorbidity scores, physicians may have used comorbidity status to restrict hyperfractionated RT to fitter individuals owing to concerns for toxicity. In the landmark Turrisi trial,^12^ radiation-induced esophagitis was significantly increased among cancer patients receiving hyperfractionated RT compared with daily RT, although these toxicity differences were not present in the more recently conducted CONVERT trial^13^ that used more modern techniques and RT target volumes (eg, no elective nodal irradiation). Notably, the CONVERT trial showed no significant survival differences between the high-dose standard fractionation treatment arm and the hyperfractionated treatment arm. Although not reaching the threshold for significance, the HR for risk of death in the standard fractionation arm was 1.18 (95% CI, 0.95-1.45; *P* = .14) compared with the hyperfractionated RT arm.^13^ Similarly, interim data from the CALGB30610 trial showed no significant differences in OS between comparable treatment schemas.^15^ The inconsistencies between our study and those from 2 randomized trials (CONVERT and CALGB 30610) may be due to patient selection and control of confounders. Although randomized trials have an obvious advantage of controlling confounders, criteria for patient enrollment are often highly selective. Our study included real-world clinical treatment outcome data. We have controlled for many covariates through multivariate adjustment and PS analyses, although the possibility of residual confounding from factors that were not or imperfectly measured cannot be completely ruled out. Further studies are required to investigate the influence of radiation dose and fractionation in treating LS-SCLC.

We also found differences in the time to RT treatment from diagnosis among RT groups. Patients with delayed RT may benefit from receiving hyperfractionated RT compared with daily fractionated subgroups. Several meta-analyses have provided evidence that quicker initiation and/or shorter duration of RT may significantly improve survival.^24^^,^^25^ Wait times from referral to the initiation of CCRT may be disproportionate among RT groups and may be modified depending on cancer network, socioeconomic status, geographic location, etc. Thus, future studies investigating the connection between RT treatment schema and treatment initiation times are warranted.

Our study has several strengths, including a study population of more than 2000 SCLC patients with comprehensive covariate information, which allowed for comprehensive adjustment for potential confounders. Stringent inclusion and exclusion criteria minimized selection biases. In addition to multivariate analyses, we performed a PS-weighted analysis. Similar results from 2 different analyses support a true association between RT dose and survival. We further included survival analyses at 1- and 3-year time intervals to evaluate changes in these associations as a function of time.

The present study has some limitations. The observational but nonrandomized nature of our study may include residual confounding. Hyperfractionated treatment entails logistical challenges beyond once-daily fractionations; thus, it may have been more frequently applied in academic and comprehensive cancer centers compared with community treatment centers. Furthermore, academic centers may offer lifestyle changes to improve outcomes, including access to dieticians or smoking cessation, that may not be available at community health centers; unfortunately, these covariates were not cataloged in the NCDB. The interactions among RT treatment group, facility type, and performance status remain an interesting caveat to our retrospective analysis and should be noted for future studies aiming to optimize RT dosing and fractionation. Additionally, comorbidity index may not accurately represent performance status. Ideally, it would be combined with other covariates such as ECOG score, body mass index, or other performance measures to strengthen the models used in our study. Furthermore, selection bias may have influenced the results. In addition, it should be noted that patients’ income and education level in the NCDB was based on neighborhood rather than individual information. Thus, residual confounding from these less well-measured covariates, as well as variables that were not measured in the NCDB (eg, body mass index, smoking status, etc.), cannot be ruled out. Another limitation is that the NCDB does not specifically record information regarding the frequency of RT being received; thus, although accounting for total dose received and treatment duration, we had to assume that all patients receiving 1.5 Gy per fraction were on a bid treatment schedule, and patients receiving either 1.8 Gy or 2.0 Gy per fraction received RT on a QD schedule. To minimize misclassification, we have excluded from the study patients whose RT doses were outside of 1.5, 1.8 and 2.0 Gy per fraction at a treatment volume between 30 and 35 fractions. Nevertheless, we cannot exclude the possibility of misclassifying patients to the wrong group. These misclassifications are likely to reduce the statistical power of our study to detect a true difference. Finally, although our study evaluated a relatively large number of SCLC patients, the sample size is still less than optimal, particularly for subgroup analyses. For example, the benefits of prophylactic cranial irradiation (PCI), as well as the use of immunotherapy in lung cancers, have been reported.^26^^,^^27^ Although these variables are recorded in the NCDB, few patients had these exposures; thus, our study lacks statistical power to analyze these patients as separate groups.

In conclusion, we observed that hyperfractionated RT was associated with improved survival in patients with LS-SCLC undergoing CCRT compared with daily RT regimens. This association was predominantly seen in stage IIb-III SCLC. Stratified analyses suggest the survival benefit may be modified by patient demographics such as age, sex, and race, as well as by time interval between diagnosis to treatment and treatment facility type. Larger, randomized controlled trials are necessary to determine dosing and dose-fractionation for optimizing RT regimens for patients with LS-SCLC undergoing CCRT.
